# Low plasma tryptophan is associated with olfactory function in healthy elderly community dwellers in Japan

**DOI:** 10.1186/s12877-017-0639-5

**Published:** 2017-10-16

**Authors:** Yusuke Adachi, Yoshiki Shimodaira, Hidehiro Nakamura, Akira Imaizumi, Maiko Mori, Yoko Kageyama, Yasushi Noguchi, Asuka Seki, Yuki Okabe, Yuko Miyake, Kaori Ono, Shu Kumagai

**Affiliations:** 1 Ajinomoto Co., Inc., Kawasaki-shi, Kanagawa 210-8681 Japan; 2 University of Human Arts and Sciences, Saitama, Saitama-shi Japan

**Keywords:** Tryptophan, Olfactory function, Plasma amino acid, Low plasma tryptophan

## Abstract

**Background:**

Decreased circulating tryptophan (Trp) levels are frequently observed in elderly patients with neurodegenerative disease including Alzheimer’s disease. Trp may serve as a potential biomarker for monitoring disease risk in elderly people. We aimed to investigate the association between low plasma Trp levels and olfactory function, which is known to predict age-related diseases including dementia in elderly people.

**Methods:**

A total of 144 healthy elderly Japanese community (≥ 65 years old) dwellers from the Health, Aging and Nutritional Improvement study (HANI study) were the subjects of our analysis. Low Trp levels were classified using the lower limit values of the reference interval according to a previous report. Olfactory function was assessed using a card-type test called Open Essence, which includes 12 odour items that are familiar to Japanese people. The elderly subjects with low circulating Trp levels were compared to a control group with normal plasma Trp levels.

**Results:**

We conducted the analyses using 144 people aged 65 years or older (mean age 73.7 ± 5.5 years; 36.1% men). The subjects showed normal serum albumin levels (4.4 ± 0.2 g/dL) and no daily living disabilities. Low plasma Trp levels (low Trp group) were found in 11.1% of the study population. The low Trp group showed a significantly lower correct-answer rate for the items india ink, perfume, curry and sweaty smelling socks than control group (*P* < 0.05). There was also a significant association between low Trp levels and low olfactory ability, after adjusting for age and sex.

**Conclusions:**

Lower plasma Trp levels were associated with a decrease in olfactory function in functionally competent older individuals. Because olfactory dysfunction predicts age-related diseases, low plasma Trp levels may represent a clinical sign of disease risk in elderly people.

**Electronic supplementary material:**

The online version of this article (10.1186/s12877-017-0639-5) contains supplementary material, which is available to authorized users.

## Background

Ageing is associated with physiological and mental decline, which leads to life-limiting conditions. Early detection of disease risk is recognized as a critical strategy for the prevention of age-related diseases and the extension of lifespan. However, few clinical biomarkers have been identified to monitor the health conditions of elderly people.

Amino acids in biological fluids provide important biochemical and nutritional information [1, 2]. Tryptophan (Trp) is an essential amino acid that plays several important roles including the regulation of neuronal activity [3]. In rodents and humans, two rate-limiting enzymes, indoleamine 2,3-dioxygenase (IDO) and tryptophan 2,3-dioxygenase (TDO), regulate systemic and local Trp levels by converting Trp to kynurenines [4]. The overexpression of these two enzymes is reported to enhance Trp degradation, which results in low Trp levels in tissues and biological fluids [3]. Because Trp is also converted into neurotransmitters such as serotonin and melatonin, enhanced Trp degradation through activation of the kynurenine pathway could lead to low levels of these neurotransmitters and affect neuronal activity. Indeed, low circulating Trp levels are frequently observed in elderly patients with neurodegenerative disease including Alzheimer’s disease [5–8]. Although the precise mechanism underlying decreased plasma Trp levels, elevated IDO expression by increased inflammatory cytokines (e.g. IFN-γ) could contribute to low plasma Trp levels in the age-related diseases. Therefore, Trp may serve as a potential biomarker for monitoring disease risk in elderly people.

Olfaction is a critical component of human physiology and is essential for the maintenance of health through avoiding environmental hazards or the regulation of appetite [9, 10]. Some independent studies have revealed that olfactory dysfunction predicts age-related diseases and mortality [11–14]. Therefore, olfactory dysfunction appears to precede the clinical signs of health problems in elderly people. The olfactory system consists of the olfactory conductions, the olfactory bulbs, and multiple brain regions including the primary olfactory cortices and higher order brain regions involved in odour recognition and memory [15]. The olfactory bulb is the initial processing centre for odorant sensory information transduced by olfactory receptor neurons in the olfactory epithelium. Interestingly, the olfactory bulb is densely innervated by serotonergic fibres [16] and expresses melatonin receptors in mammals [17]. Because Trp is a precursor for these neurotransmitters, we have hypothesized that abnormal Trp metabolism might be associated with olfactory dysfunction.

Recently, we performed a community-based cohort study called the Health, Aging and Nutritional Improvement study (HANI study), which was designed to investigate the relationships between nutrition, food, and health conditions. Olfactory function was included in the HANI study to assess the association between disease risk and plasma amino acid profiles. Here, we analysed the baseline data from the HANI study and found associations between low plasma Trp levels and olfactory dysfunction in elderly people.

## Methods

### Subjects

The HANI study is an interventional study addressing the relationship between life style and health conditions that includes a five-year follow-up to determine disease risk. Briefly, a total of 330 community-dwelling adults age 60 or older residing in Kita-ku, Tokyo were recruited in November in 2013. Participants annually underwent the following procedures: 1) venous blood draw, 2) extensive physical and olfactory examination, and 3) questionnaire regarding dietary history. In February 2014, a modest intervention of nutrition counselling was started and continued until March 2016. In this paper, we analysed the baseline data from 2013 before the intervention to examine relationships between plasma amino acids, biochemical data and olfactory function. The plasma amino acid levels of 144 elderly people (65 years or older) were subjected to analysis. All data were analysed anonymously throughout the study.

### Clinical assessment

Body composition (body weight, body fat mass and skeletal muscle mass) was determined using bioelectrical impedance analysis (Inbody 2.0, InBody Japan, Inc., Tokyo, Japan). Obese and lean elderly subjects were identified using a body mass index (BMI) ≥ 25 and <18.5, respectively. Protein malnutrition was defined as albumin levels <3.5 g/L. Anaemia was determined using the World Health Organization (WHO) criteria (haemoglobin (Hb) levels <13 g/dL for men and <12 g/dL for women). Dietary intake was estimated using a brief-administered dietary history questionnaire (BDHQ) for the subjects [18]. The BDHQ was conducted on the same day as blood draw and olfactory examination.

### Measurement of olfactory function

Odour identification was assessed using the Open Essence test (OE test, Wako Pure Chemical Industries, Saitama, Japan) [19, 20]. The OE test is a card-type test developed for Japanese people to assess odour identification. The OE test includes 12 odour items that are familiar to Japanese people (india ink, timber, perfume, menthol, orange, curry, cooking gas, rose, the Japanese cypress “hinoki”, sweaty smelling socks, condensed milk, and roasted garlic). Subjects wiped their fingers with an odourless wet tissue to remove any smells before the OE test. The OE score was defined as the number of correct answers [21]. Scores of 7 or lower on the OE test were defined as olfactory dysfunction [21].

### Determination of amino acid levels in plasma

Blood samples were taken after an overnight fast. Blood samples were collected from the forearm veins of subjects and stored in tubes containing disodium ethylenediaminetetraacetate (2Na·EDTA). The tubes were immediately placed in a portable blood tube cooler (CubeCooler®; Tochigi, Japan). Plasma was prepared by centrifuging the samples at 3000 rpm at 4 °C for 15 min and then stored at −80 °C until analysis. The plasma samples were deproteinized using acetonitrile at a final concentration of 80% before the measurements. The concentrations of the plasma amino acids were measured using HPLC–ESI–MS, followed by pre-column derivatization using previously described analytical methods [22]. The concentrations of essential amino acids are listed in Additional file 1: Table S1. Individuals with low plasma amino acid levels were classified according to the lower limit values (men: 47 μg/mL; women: 40.9 μg/mL) of the reference interval from a recent report [23].

### Statistical analysis

Categorical data were analysed using Fisher’s exact test (JMP ver. 12.1.1 software, SAS Institute Inc., Cary, NC, USA), and continuous data were compared using Welch’s *t*-tests (GraphPad Prism ver. 6.07, GraphPad Software, La Jolla, CA, USA). The association of low plasma Trp levels with olfactory dysfunction was examined using multiple logistic regression models adjusting for age and sex (likelihood ratio tests: JMP ver. 12.1.1 software, SAS Institute Inc., Cary, NC, USA). Significance was set at *P* < 0.05. All data were analysed anonymously throughout the study.

## Results

### Characteristics of the elderly subjects in the HANI study

The population characteristics of the elderly subjects in the HANI study are summarized in Table 1. There were no subjects with abnormal biochemical parameters, such as serum albumin or impaired activities of daily living (ADL). The prevalence of anaemia was 6.5%. The plasma amino acid levels were determined, and the number of subjects with low plasma free amino acid levels were calculated based on the concentrations of the plasma free amino acids shown in Additional file 1: Table S1. Among the essential amino acids, the highest rate was observed in the elderly subjects with low plasma Trp levels (low Trp group, 11.1%). The low Trp group was compared with the control group with normal plasma Trp levels. The amino acid profile of the low Trp group is shown in Additional file 2: Table S2. There were no significant differences in BMI, serum albumin, and haemoglobin between the low Trp group and the normal Trp group. The mean age in the low Trp group was slightly higher than in the normal Trp group (Table 1). There were no significant differences in energy intake and protein intake between normal and low Trp group (Additional file 3: Table S3).

**Table 1:** Comparison of age, body mass index (BMI), biochemical parameters, abilities of daily living (ADL) and olfactory and health characteristic in the study population

Variable	Total	Normal Trp group	Low Trp group
Number (% of men)	144 (36.1)	128 (36.7)	16 (31.3)
Age, y (range)	73.7 ± 5.5 (65−93)	73.2 ± 5.3 (65−93)	77.5 ± 6.4 (66−91)*
BMI, kg/m^2^ (range)	23.4 ± 3.2 (16.7−33.6)	23.4 ± 3.0 (16.9−33.6)	23.7 ± 4.3 (16.7−31.7)
Serum albumin, g/dL (range)	4.4 ± 0.2 (3.7−4.9)	4.4 ± 0.2 (3.7−4.9)	4.4 ± 0.2 (4.0−4.9)
Hemoglobin, mg/dL (range)	13.9 ± 1.3 (10.1−16.7)	13.9 ± 1.3 (10.1−16.7)	13.5 ± 1.3 (11.5−15.9)
CRP, mg/L (range)	0.090 ± 0.10 (0.004−0.5)	0.095 ± 0.11 (0.007−0.5)	0.055 ± 0.04 (0.004−0.143)**
Low albumin, % (n)	None	None	None
Anemia, % (n)	6.3 (9)	5.5 (7)	12.5 (2)
High CRP, % (n)	None	None	None
ADL disability	None	None	None
OE score	6.2 ± 2.9 (0−12)	6.5 ± 2.8 (0−12)	4.4 ± 2.4 (0−8)**

OE score: the open essence score; None: no subjects. Data are expressed as mean ± SD. Significant: *P < 0.05, **P < 0.01 between normal Trp and low Trp group (Welch’s t-tests or Fisher’s exact test)

### Relationship between low plasma Trp levels and olfactory function

Next, we examined the relationship between olfactory function and low plasma Trp levels. The mean OE score of the low Trp group was lower than that of the control group (4.4 ± 2.4 vs. 6.5 ± 2.8, *P* = 0.0049, Table 1). The low Trp group showed a higher rate of olfactory dysfunction compared with the control group (94% vs. 61%; odds ratio [OR], 9.62; 95% CI, 1.2–75.1; *P* = 0.0107).

The correct-answer rate for each smell in the OE test is listed in Table 2. The low Trp group showed a significantly lower correct-answer rate for india ink (25.0% vs. 63.0%; OR, 5.11; 95% CI, 1.6–16.7; *P* = 0.0058), perfume (6.3% vs. 35.9%; OR, 8.41; 95% CI, 1.1–65.8; *P* = 0.0211), curry (62.5% vs. 84.4%; OR, 3.24; 95% CI, 1.1–9.9; *P* = 0.0431) and sweaty smelling socks (31.3% vs. 63.3%; OR, 3.79; 95% CI, 1.2–11.6; *P* = 0.0277) compared with the control group. There were no significant differences in the correct-answer rate for timber, menthol, mandarin orange, cooking gas, rose, cypress wood and roasted garlic between the two groups. Because the low Trp group (77.5 ± 6.4 years) was slightly older than the control group (73.2 ± 5.3 years), a multiple regression analysis was performed to consider the factor of age. Even after adjusting for age and sex, significant associations remained between low plasma Trp levels and a low correct-answer rate for India ink (OR, 5.16; 95% CI, 1.6–19.9; *P* = 0.0047), perfume (OR, 7.40; 95% CI, 1.38–137; *P* = 0.0154) and sweaty smelling socks (OR, 3.26; 95% CI, 1.06–11.2; *P* = 0.0384) in the OE test (Table 3). There was also a significant association between low Trp levels and olfactory dysfunction in the adjusted analysis (OR, 7.75; 95% CI 1.42–145, *P* = 0.0142). On the other hand, there was no significant relationship between low plasma concentrations of other essential amino acids (e.g. leucine) and olfactory dysfunction (data not shown). These results suggest that low plasma Trp levels are independently associated with olfactory dysfunction.

**Table 2:** The score for each smell in the OE tests in the normal Trp group and low Trp group

Smell	Correct answer rate (%)		
	Normal	Low Trp	Significance
India ink	63.0	25.0	**
Timber	29.7	37.5	N.S.
Perfume	35.9	6.3	*
Menthol	63.2	68.8	N.S.
Mandarin orange	57.0	43.8	N.S.
Curry	84.4	62.5	*
Gas	44.9	25.0	N.S.
Rose	28.9	6.3	N.S.
Cypress	65.6	43.8	N.S.
Sweaty smelling socks	63.3	31.3	*
Condensed milk	66.4	60.0	N.S.
Fried garlic	46.1	31.3	N.S.

Each column shows the correct answer rate for each group. Significant: *P < 0.05, **P < 0.01 between normal Trp and low Trp group (Fisher’s exact test)

**Table 3:** Effects of low plasma Trp level on olfactory functions in the study population

Variable	Odds ratio (95% CI)	*P* value
India ink	5.16 (1.6–19.9)	0.0047**
Perfume	7.40 (1.38–137)	0.0154*
Curry	3.02 (0.84–10.2)	0.0871
Sweaty smelling socks	3.26 (1.06–11.2)	0.0384*
OE score ≤ 7	7.75 (1.42–145)	0.0142*

Odds ratio for correct answer to each smell or olfactory function (OE score < 8) was adjusted by age and sex. 95% CI: 95% Confidence interval. Significant: *P < 0.05; **P < 0.01 (likelihood ratio tests)

## Discussion

Trp is a precursor of several bioactive compounds, including neurotransmitters, and its metabolism plays a crucial role in the immune system and neuronal activity. In fact, enhanced degradation of Trp through the kynurenine pathway has been observed in several diseases including immunological disorders [24, 25] or neurodegenerative diseases (e.g., Alzheimer’s disease) [5, 6, 8]. Therefore, low circulating Trp levels could be associated with diseases and health problems. In the present study, we found an association between low plasma Trp levels and olfactory dysfunction in Japanese elderly people. This result is tempting to speculate that low circulating Trp levels reflect abnormal Trp metabolism, which leads to the dysregulation of bioactive Trp metabolites and consequently causes olfactory dysfunction. Thus, low levels of plasma Trp may indicate decreased olfactory function.

Potentially modifiable lifestyle factors could reduce the risk of cognitive decline and neurodegenerative diseases in elderly individuals. In recent years, the concept of cognitive reserve has been proposed as a mechanism to explain individual differences in rates of cognitive decline [26]. It is possible that lifestyle factors may enhance cognitive reserve, resulting in greater resilience against the effects of developing neuropathology [27, 28]. Interestingly, one study reported that olfactory function may act as a cognitive reserve in patients with Parkinson’s disease [29]. Although the underlying mechanism is still unclear, olfactory dysfunction may interfere with life style factors such as dietary patterns and thus affect cognitive reserve [26, 28], which may in turn explain how olfactory dysfunction predicts neurodegenerative diseases and mortality [11–14]. Because low plasma Trp levels are associated with olfactory dysfunction, we hypothesize that low plasma Trp levels represent a clinical sign of disease risk or reduced cognitive reserve in elderly individuals. Further longitudinal analysis will be required to definitively address this hypothesis.

There are three major limitations of this study. First, the number of elderly subjects with low plasma Trp levels was small. Although the rate of elderly people with low plasma Trp levels was the largest (11.1%) among the essential amino acids, further clinical studies are needed with a larger sample size. Second, only the OE test was used to assess olfactory function. We chose this method in our study because the OE test consists of odorants familiar to the Japanese population and has been used for several studies in Japanese subjects (20). However, numerous tests using various types of smells are available to assess olfactory function (e.g., the University of Pennsylvania Smell Identification Test: UPSIT). In the present study, the scores for the smells of India ink, perfume, and cypress wood were significantly lower in elderly people with low plasma Trp levels, while other smells showed no difference between the groups (Table 3). This clearly indicates that clinical studies may produce different results depending on the methods used to assess olfactory function. Thus, other methods should be considered for future studies. Last, because of the cross-sectional analysis, we did not demonstrate a direct association between low Trp levels and olfactory dysfunction. However, although there has been no evidence for the effects of supplementation of Trp alone on olfactory function, a combination of DHA, melatonin and Trp was shown to improve olfactory function in patients with mild cognitive impairment (MCI) [30], which supports our hypothesis that Trp dysregulation affects olfactory function.

## Conclusions

Our findings showed that low plasma Trp levels were associated with olfactory dysfunction in healthy elderly community dwellers in Japan. Thus, low levels of plasma Trp may lead to a decrease in olfactory function. Because there is considerable evidence that olfactory function contributes to the pathophysiology or development of diseases, plasma Trp may serve as a potential marker of health problems or disease risk in elderly populations.

## Additional files

Additional file 1: Table S1. Concentrations of the rate of elderly subjects with low essential amino acid levels and plasma essential amino acid levels in the study. (DOCX 16 kb)

Additional file 2: Table S2. The concentrations of plasma amino acid and related metabolites of the normal Trp group and the low Trp group. (DOCX 20 kb)

Additional file 3: Table S3. Concentrations of the rate of elderly subjects with low essential amino acid levels and plasma essential amino acid levels in the study population. (DOCX 14 kb)

## Abbreviations

ADL: Activities of daily living
EDTA: Ethylenediaminetetraacetate
HANI study: The Health, Aging and Nutritional Improvement study
IDO: Indoleamine 2,3-dioxygenase
MCI: Mild Cognitive Impairment
OE test: Open Essence test
TDO: Tryptophan 2,3-dioxygenase
Trp: Tryptophan
UPSIT: The University of Pennsylvania Smell Identification Test

## References

[1] Yamakado M, Nagao K, Imaizumi A, Tani M, Toda A, Tanaka T, Jinzu H, Miyano H, Yamamoto H, Daimon T (2015). Plasma free amino acid profiles predict four-year risk of developing diabetes, metabolic syndrome, dyslipidemia, and hypertension in Japanese population. Sci Rep.

[2] Noguchi Y, Zhang QW, Sugimoto T, Furuhata Y, Sakai R, Mori M, Takahashi M, Kimura T (2006). Network analysis of plasma and tissue amino acids and the generation of an amino index for potential diagnostic use. Am J Clin Nutr.

[3] van der Goot AT, Nollen EA (2013). Tryptophan metabolism: entering the field of aging and age-related pathologies. Trends Mol Med.

[4] Stone TW, Darlington LG (2002). Endogenous kynurenines as targets for drug discovery and development. Nat Rev Drug Discov.

[5] Widner B, Leblhuber F, Walli J, Tilz GP, Demel U, Fuchs D (2000). Tryptophan degradation and immune activation in Alzheimer's disease. J Neural Transm (Vienna).

[6] Fekkes D, van der Cammen TJ, van Loon CP, Verschoor C, van Harskamp F, de Koning I, Schudel WJ, Pepplinkhuizen L (1998). Abnormal amino acid metabolism in patients with early stage Alzheimer dementia. J Neural Transm (Vienna).

[7] Trushina E, Dutta T, Persson XM, Mielke MM, Petersen RC (2013). Identification of altered metabolic pathways in plasma and CSF in mild cognitive impairment and Alzheimer's disease using metabolomics. PLoS One.

[8] Widner B, Leblhuber F, Walli J, Tilz GP, Demel U, Fuchs D (1999). Degradation of tryptophan in neurodegenerative disorders. Adv Exp Med Biol.

[9] Mennella JA, Jagnow CP, Beauchamp GK (2001). Prenatal and postnatal flavor learning by human infants. Pediatrics.

[10] Hays NP, Roberts SB (2006). The anorexia of aging in humans. Physiol Behav.

[11] Wilson RS, Yu L, Bennett DA (2011). Odor identification and mortality in old age. Chem Senses.

[12] Pinto JM, Wroblewski KE, Kern DW, Schumm LP, McClintock MK (2014). Olfactory dysfunction predicts 5-year mortality in older adults. PLoS One.

[13] Eibenstein A, Fioretti AB, Simaskou MN, Sucapane P, Mearelli S, Mina C, Amabile G, Fusetti M (2005). Olfactory screening test in mild cognitive impairment. Neurol Sci.

[14] Wesson DW, Wilson DA, Nixon RA (2010). Should olfactory dysfunction be used as a biomarker of Alzheimer's disease?. Expert Rev Neurother.

[15] Kovacs T (2004). Mechanisms of olfactory dysfunction in aging and neurodegenerative disorders. Ageing Res Rev.

[16] Dugue GP, Mainen ZF (2009). How serotonin gates olfactory information flow. Nat Neurosci.

[17] Corthell JT, Olcese J, Trombley PQ (2014). Melatonin in the mammalian olfactory bulb. Neuroscience.

[18] Okubo H, Sasaki S, Rafamantanantsoa HH, Ishikawa-Takata K, Okazaki H, Tabata I (2008). Validation of self-reported energy intake by a self-administered diet history questionnaire using the doubly labeled water method in 140 Japanese adults. Eur J Clin Nutr.

[19] Okutani F, Hirose K, Kobayashi T, Kaba H, Hyodo M (2013). Evaluation of "open essence" odor-identification test card by application to healthy volunteers. Auris Nasus Larynx.

[20] Makizako M, Makizako H, Doi T, Uemura K, Tsutsumimoto K, Miyaguchi H, Shimada H (2014). Olfactory identification and cognitive performance in community-dwelling older adults with mild cognitive impairment. Chem Senses.

[21] Fujio H, Doi K, Hasegawa S, Kobayakawa T, Nibu K (2012). Evaluation of card-type odor identification test for Japanese patients with olfactory disturbance. Ann Otol Rhinol Laryngol.

[22] Shimbo K, Kubo S, Harada Y, Oonuki T, Yokokura T, Yoshida H, Amao M, Nakamura M, Kageyama N, Yamazaki J (2010). Automated precolumn derivatization system for analyzing physiological amino acids by liquid chromatography/mass spectrometry. Biomed Chromatogr.

[23] Yamamoto H, Kondo K, Tanaka T, Muramatsu T, Yoshida H, Imaizumi A, Nagao K, Noguchi Y, Miyano H (2016). Reference intervals for plasma-free amino acid in a Japanese population. Ann Clin Biochem.

[24] Denz H, Orth B, Weiss G, Herrmann R, Huber P, Wachter H, Fuchs D (1993). Weight loss in patients with hematological neoplasias is associated with immune system stimulation. Clin Investig.

[25] Iwagaki H, Hizuta A, Tanaka N, Orita K (1995). Decreased serum tryptophan in patients with cancer cachexia correlates with increased serum neopterin. Immunol Investig.

[26] Duffy VB, Backstrand JR, Ferris AM (1995). Olfactory dysfunction and related nutritional risk in free-living, elderly women. J Am Diet Assoc.

[27] Freret T, Gaudreau P, Schumann-Bard P, Billard JM, Popa-Wagner A (2015). Mechanisms underlying the neuroprotective effect of brain reserve against late life depression. J Neural Transm (Vienna).

[28] Clare L, YT W, Teale JC, MacLeod C, Matthews F, Brayne C, Woods B, Team CF-Ws (2017). Potentially modifiable lifestyle factors, cognitive reserve, and cognitive function in later life: a cross-sectional study. PLoS Med.

[29] Lee JE, Cho KH, Ham JH, Song SK, Sohn YH, Lee PH (2014). Olfactory performance acts as a cognitive reserve in non-demented patients with Parkinson's disease. Parkinsonism Relat Disord.

[30] Rondanelli M, Opizzi A, Faliva M, Mozzoni M, Antoniello N, Cazzola R, Savare R, Cerutti R, Grossi E, Cestaro B (2012). Effects of a diet integration with an oily emulsion of DHA-phospholipids containing melatonin and tryptophan in elderly patients suffering from mild cognitive impairment. Nutr Neurosci.

