# Supplementary material for: Low plasma tryptophan is associated with olfactory function in healthy elderly community dwellers in Japan
Source: BMC Geriatr. 2017 Oct 16;17:239. doi: 10.1186/s12877-017-0639-5 (PMC5644149; doi:10.1186/s12877-017-0639-5)
Supplement: Supplementary file 1 — Concentrations of the rate of elderly subjects with low essential amino acid levels and plasma essential amino acid levels in the study. (DOCX 16 kb) [file 12877_2017_639_MOESM1_ESM.docx]

**Table S1**. Concentrations of the rate of elderly subjects with low essential amino acid levels and plasma essential amino acid levels in the study population.

| Rate Ranking | Under the cutoff  % (n) | Plasma concentration, μM Mean ± SD (range) |
| --- | --- | --- |
| Trp | 11.1 (16) | 51.6 ± 9.0 (29.6−87.5) |
| Leu | 6.9 (10) | 115.5 ± 19.7 (68.8−182.2) |
| Val | 3.5 (5) | 216.6 ± 35.8 (144.2−344.3) |
| His | 2.1 (3) | 80.1 ± 9.5 (62.4−121.1) |
| Thr | 2.1 (3) | 117.7 ± 25.2 (75.6−215.1) |
| Ile | 1.4 (2) | 59.5 ± 11.5 (37.1−103.9) |
| Met | 0.7 (1) | 25.6 ± 4.8 (17.4−42.9) |
| Phe | 0.7 (1) | 59.4 ± 8.5 (39.5−94.8) |
| Lys | None | 191.3 ± 28.4 (134.2−271.5) |

The subjects with low plasma amino acid levels were classified using lower limit values in recently reported reference intervals (23)
