# Supplementary material for: Low plasma tryptophan is associated with olfactory function in healthy elderly community dwellers in Japan
Source: BMC Geriatr. 2017 Oct 16;17:239. doi: 10.1186/s12877-017-0639-5 (PMC5644149; doi:10.1186/s12877-017-0639-5)
Supplement: Supplementary file 2 — The concentrations of plasma amino acid and related metabolites of the normal Trp group and the low Trp group. (DOCX 20 kb) [file 12877_2017_639_MOESM2_ESM.docx]

**Table S2.** The concentrations of plasma amino acid and related metabolites of the normal Trp group and the low Trp group.

| Variable | Men | | *P* value | Women | | *P* value |
| --- | --- | --- | --- | --- | --- | --- |
|  | Normal Trp (n = 47) | Low Trp (n =5) |  | Normal Trp (n = 81) | Low Trp (n = 11) |  |
| Non-essential amino and the related metabolites | | | | | | |
| Glycine | 207 ± 33 (148−280) | 216 ± 27 (175−240) | 0.5388 | 232 ± 69 (137−441) | 261 ± 100 (170−481) | 0.3688 |
| Alanine | 375 ± 84 (150−592) | 358 ± 34 (319−396) | 0.3849 | 350 ± 78 (212−553) | 302 ± 72 (198−432) | 0.0598 |
| Proline | 152 ± 39 (93−258) | 158 ± 25 (127−191) | 0.6755 | 128 ± 34 (71−227) | 145 ± 96 (61−392) | 0.5628 |
| Serine | 106 ± 15 (52−152) | 102 ± 9 (90−110) | 0.3624 | 107 ± 19 (70−165) | 112 ± 27 (74−161) | 0.5691 |
| Asparagine | 48.2 ± 7.3 (35.0−62.8) | 48.6 ± 7.0 (38.4−57.0) | 0.9092 | 45.8 ± 8.9 (33.3−89.5) | 42.0 ± 6.4 (35.2−56.3) | 0.0985 |
| Glutamine | 628 ± 77 (517−849) | 687 ± 112 (569−845) | 0.3070 | 611 ± 64 (464−780) | 581 ± 45 (533−669) | 0.0664 |
| Glutamic acid | 27.4 ± 9.8 (9.0−47.7) | 24.9 ± 11.4 (11.0−42.1) | 0.6586 | 24.1 ± 11.9 (9.1−63.1) | 22.4 ± 11.0 (8.1−42.8) | 0.6451 |
| Tyrosine | 70.7 ± 10.6 (52.3−101.6) | 66.4 ± 12.8 (52.7−85.4) | 0.5058 | 66.0 ± 10.6 (45.8−102.8) | 55.3 ± 10.0 (41.8−71.8) | 0.0056* |
| Ornithine | 60.2 ± 13.5 (40.3−114.5) | 52.6 ± 5.5 (48.6−60.6) | 0.0340* | 54.6 ± 12.3 (29.4−100.4) | 59.4 ± 18.3 (37.9−94.8) | 0.4199 |
| Citrulline | 38.1 ± 8.6 (24.8−60.3) | 50.7 ± 13.9 (34.6−68.1) | 0.1116 | 37.0 ± 8.4 (17.9−58.9) | 38.9 ± 14.1 (19.3−73.8) | 0.6639 |
| Arginine | 103 ± 20 (68−162) | 108 ± 13 (93−118) | 0.4473 | 99 ± 17 (63−139) | 88 ± 12 (62−112) | 0.0135* |
| α-Aminobutyric acid | 19.4 ± 5.5 (8.2−35.9) | 16.8 ± 4.8 (11.5−21.4) | 0.3049 | 18.0 ± 4.8 (8.1−28.8) | 16.2 ± 5.4 (9.7−25.9) | 0.3258 |
| Essential amino acid |  |  |  |  |  |  |
| Threonine | 124 ± 22 (86−191) | 114 ± 41 (76−176) | 0.5872 | 117 ± 26 (77−215) | 98 ± 16 (80−130) | 0.0040** |
| Histidine | 83.9 ± 10.3 (65.9−121.1) | 85.3 ± 11.7 (67.8−99.8) | 0.8001 | 78.2 ± 8.3 (62.4−104.7) | 75.5 ± 7.4 (62.7−84.1) | 0.2797 |
| Methionine | 27.7 ± 5.3 (19.1−42.9) | 30.3 ± 5.9 (22.5−39.0) | 0.3809 | 24.5 ± 3.5 (17.4−35.2) | 22.9 ± 5.8 (18.8−38.5) | 0.3954 |
| Lysine | 201 ± 27 (152−253) | 183 ± 18 (162−209) | 0.0973 | 189 ± 29 (134−272) | 170 ± 19 (149−214) | 0.0100* |
| Valine | 234 ± 41 (159−344) | 200 ± 24 (175−234) | 0.0285* | 210 ± 30 (147−271) | 201 ± 34 (144−260) | 0.4621 |
| Isoleucine | 65.8 ± 13.5 (43.4−103.9) | 64.6 ± 9.4 (55.7−79.0) | 0.7955 | 56.3 ± 8.3 (41.3−77.7) | 54.1 ± 11.8 (37.1−74.5) | 0.5635 |
| Leucine | 128 ± 22 (92−182) | 109 ± 18 (86−133) | 0.0806 | 111 ± 15 (69−142) | 99 ± 16 (72−125) | 0.0349* |
| Phenylalanine | 62.8 ± 9.7 (47.0−94.8) | 64.3 ± 8.2 (54.6−74.0) | 0.7274 | 57.9 ± 6.8 (43.2−86.5) | 53.5 ± 9.3 (39.5−71.0) | 0.1546 |
| Tryptophan | 58.2 ± 8.0 (48.1−87.5) | 42.4 ± 4.6 (35.1−46.2) | 0.0003*** | 50.5 ± 6.3 (41.0−72.8) | 36.0 ± 3.8 (29.6−40.9) | < 0.001*** |

Data are expressed as mean ± SD. Significant: **P* < 0.05, ***P* < 0.01, ****P* < 0.001 between normal Trp and low Trp group (Welch’s t-tests)
