# Supplementary material for: Low plasma tryptophan is associated with olfactory function in healthy elderly community dwellers in Japan
Source: BMC Geriatr. 2017 Oct 16;17:239. doi: 10.1186/s12877-017-0639-5 (PMC5644149; doi:10.1186/s12877-017-0639-5)
Supplement: Supplementary file 3 — Concentrations of the rate of elderly subjects with low essential amino acid levels and plasma essential amino acid levels in the study population. (DOCX 14 kb) [file 12877_2017_639_MOESM3_ESM.docx]

**Table S3**. Comparison of nutrient consumption between the normal Trp group and the low Trp group

| Nutrient consumption | Normal Trp | Low Trp | P value |
| --- | --- | --- | --- |
| Energy (kcal) | 2062 ± 622 | 2241 ± 662 | 0.3174 |
| Protein (g/1000 kcal) | 42.9 ± 8.4 | 46.3 ± 8.2 | 0.1319 |
| Animal protein (g/1000 kcal) | 26.3 ± 8.8 | 29.5 ± 8.4 | 0.1664 |
| Vegetable protein (g/1000 kcal) | 16.6 ± 2.8 | 16.8 ± 2.7 | 0.7653 |
| Carbohydrate (g/1000 kcal) | 128 ± 17 | 124 ± 20 | 0.5312 |
| Fat (g/1000 kcal) | 31.2 ± 5.8 | 33.5 ± 6.1 | 0.1606 |

Data are expressed as mean ± SD. Significant: Statistical differences between normal Trp and low Trp group was tested using Welch’s t-tests.
